# Cytoprotective Effect of Epigallocatechin Gallate (EGCG)-5′-*O*-α-Glucopyranoside, a Novel EGCG Derivative

**DOI:** 10.3390/ijms19051466

**Published:** 2018-05-15

**Authors:** Sang Yun Han, Eunji Kim, Kyeonghwan Hwang, Zubair Ahmed Ratan, Hyunsik Hwang, Eun-Mi Kim, Doman Kim, Junseong Park, Jae Youl Cho

**Affiliations:** 1 Department of Integrative Biotechnology and Biomedical Institute for Convergence (BICS), Sungkyunkwan University, Suwon 16419, Korea; dangsukr@naver.com (S.Y.H.); im144069@gmail.com (E.K.); hhhyun@gmail.com (H.H.); 2 Basic Research & Innovation Division, Amorepacific Corporation R&D Center, Yongin 17074, Korea; khhwang@amorepacific.com (K.H.), emkim@amorepacific.com (E.-M.K.); 3 Department of Biomedical Engineering, Khulna University of Engineering and Technology, Khulna 9203, Bangladesh; ratandmck62@gmail.com; 4 Graduate School of International Agricultural Technology, Seoul National University, Pyeongchang 25354, Korea; kimdm@snu.ac.kr; 5 Department of Engineering Chemistry, Chungbuk National University, Cheongju 28644, Korea

**Keywords:** epigallocatechin gallate derivate, antioxidant, free radicals, cell survival, apoptosis

## Abstract

Epigallocatechin gallate (EGCG) is a well-studied polyphenol with antioxidant effects. Since EGCG has low solubility and stability, many researchers have modified EGCG residues to ameliorate these problems. A novel EGCG derivative, EGCG-5′-*O*-α-glucopyranoside (EGCG-5′Glu), was synthesized, and its characteristics were investigated. EGCG-5′Glu showed antioxidant effects in cell and cell-free systems. Under SNP-derived radical exposure, EGCG-5′Glu decreased nitric oxide (NO) production, and recovered ROS-mediated cell viability. Moreover, EGCG-5′Glu regulated apoptotic pathways (caspases) and cell survival molecules (phosphoinositide 3-kinase (PI3K) and phosphoinositide-dependent kinase 1 (PDK1)). In another radical-induced condition, ultraviolet B (UVB) irradiation, EGCG-5′Glu protected cells from UVB and regulated the PI3K/PDK1/AKT pathway. Next, the proliferative effect of EGCG-5′Glu was examined. EGCG-5′Glu increased cell proliferation by modulating nuclear factor (NF)-κB activity. EGCG-5′Glu protects and repairs cells from external damage via its antioxidant effects. These results suggest that EGCG-5′Glu could be used as a cosmetics ingredient or dietary supplement.

## 1. Introduction

Reactive oxygen species (ROS) are generated from ultraviolet B (UV) exposure, air pollution, chemical substances, and hypoxia [1,2], and can damage cells and tissues. At low concentrations of ROS, they physiologically play a role as redox messengers in signaling. However, high levels of ROS promote apoptosis and cause oxidative modification of proteins, lipids, and DNA [3,4]. ROS are involved in apoptotic signal pathways, and accumulation of ROS can induce apoptosis [4,5]. Antioxidants eliminate harmful ROS and protect the body from ROS-induced damage [6].

The epidermis is a self-renewing stratified epithelium that differentiates from the basal layer, while keratinocytes are lost from the outermost layer. Through this process, the epidermis remains in balance [7]. This maturation process is a special form of apoptosis called metabolically dead keratinized epidermal differentiation [8]. In addition, apoptotic programs and keratinocyte differentiation share signal mechanisms [9]. Protein kinase B (AKT) is the important molecule involved in the mechanisms of cell survival and proliferation. This molecule is downstream of PI3K and activated to induce apoptosis-related caspases and nuclear factor (NF)-κB signal molecules [10]. Phosphorylated AKT directly inhibits the activities of forkhead box O (FoxO) and Bcl-2-associated death promoter (BAD), known as pro-apoptotic proteins and promotes cell survival through indirect effects on p53 and NF-κB and stimulation of glucose metabolism and protein synthesis [11,12]. In most cases, activation of NF-κB depends on phosphorylation of the inhibitor of κB kinase (IKK) complex and degradation of IκB. AKT has been shown to modulate IKK activity directly and indirectly. This leads to nuclear translocation and activation of NF-κB, and transcription of the NF-κB-dependent pro-survival pathway including intrinsic-apoptosis genes (caspases-3, -7, and -9) and extrinsic-apoptosis genes (caspases-3, -8, and -10) [13,14,15]. Apoptosis is involved in several biological events including embryogenesis, tissue homeostasis, development, and aging [16,17,18,19,20,21]. For these reasons, inhibition of apoptosis is regarded as an anti-aging method [22,23].

**Figure 1:**
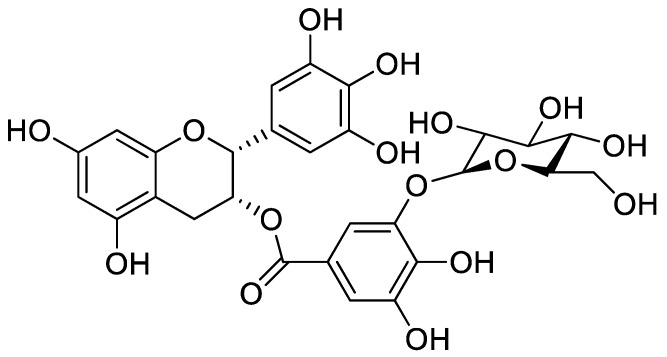
Structure of EGCG-5′-*O*-α-glucopyranoside (EGCG-5′Glu).

Epigallocatechin gallate (EGCG, (−)-epigallacatechin-3-gallate) is an abundant polyphenol in green tea (*Camellia sinesis* L. Ktze). EGCG has antioxidant, anti-inflammatory, anti-proliferative, and anti-apoptotic effects [24,25,26]. However, EGCG has limited use due to its low solubility and stability [23,27,28]. Several researchers have developed EGCG derivatives to improve these characteristics [29,30,31]. The novel EGCG derivative, EGCG-5′-*O*-α-glucopyranoside (EGCG-5′Glu, Figure 1), was previously made by attaching with glucopyranoside using dextransucrase from *Leuconostoc mesenteroide*. EGCG-5′Glu is an EGCG-glucoside and is glycosylated at the B-ring of EGCG. Kim et al. [32] showed that EGCG-5′Glu exhibited higher hydrophilic and browning-resistance properties than EGCG. However, the biological function of EGCG-5′Glu is not fully understood.

In this study, we investigated the biological properties of EGCG-5′Glu, such as its antioxidant and anti-apoptotic effects. The regulatory mechanism of EGCG-5′Glu in antioxidant-mediated apoptosis was revealed.

## 2. Results

### 2.1. Antioxidant Effect of EGCG-5′Glu in Cell and Cell-free Systems

**Figure 2:**
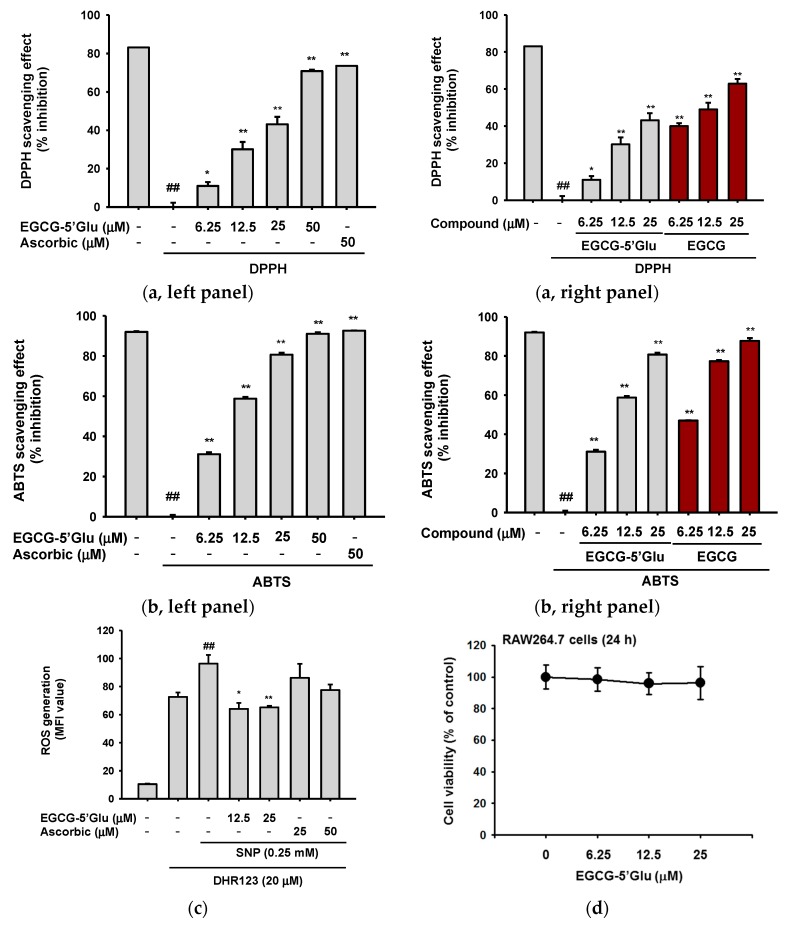
Antioxidant effect of EGCG-5′Glu. 250 mM DPPH solution was incubated with EGCG-5′Glu (0–25 μM), ascorbic acid (50 μM) (**a**, **left panel**), or EGCG (0–25 μM) (**a**, **right panel**) at 37 °C for 30 min. Absorbance at 517 nm was measured by spectrophotometry. Ascorbic acid was used as a positive control. Mixture of 2,2'-azino-bis(3-ethylbenzothiazoline-6-sulphonic acid (ABTS) solution and EGCG-5′Glu (**b**, **left panel**) or EGCG (**b**, **right panel**) was reacted at 37 °C for 30 min. Scavenging of ABTS was determined by measuring absorbance at 730 nm. Ascorbic acid was used as positive control. (**c**) DHR123 was treated on RAW264.7 cells for 10 min, and EGCG-5′Glu or ascorbic acid were then added. Cells were incubated with sodium nitroprusside (SNP) for 20 min, and reactive oxygen species (ROS) generation was determined by fluorescence-activated cell sorting (FACS). Ascorbic acid and EGCG were used for a positive control in DPPH assay, ABTS assay, and ROS generation experiment. (**d**) Cytotoxicity of EGCG-5′Glu on RAW264.7 cells was tested by MTT assay. ## *p* < 0.01 versus a normal group (untreated group), * *p* < 0.05 and ** *p* < 0.01 versus a control group (induced group).

EGCG is noted for its antioxidant effects and has been used as a positive control in antioxidant studies [33,34,35]. We examined the antioxidant effect of EGCG-5′Glu using 1,1-diphenyl-2-picrylhydrazyl (DPPH) and 2,2'-azino-bis(3-ethylbenzothiazoline-6-sulphonic acid (ABTS) assays. EGCG-5′Glu showed a scavenging effect in the DPPH assay (Figure 2a, left panel). In the ABTS assay, EGCG-5′Glu significantly cleared ABTS radicals at 25 μM (Figure 2b, left panel). The IC_50_ values of each scavenging assay were 41.80 ± 13.51 μM and 12.41 ± 1.42 μM, respectively. In addition, the antioxidative activities of EGCG-5′Glu were proved to be weaker than those of EGCG (Figure 2a, right panel, and Figure 2b, right panel). Meanwhile, the scavenging activities of EGCG-5′Glu at 50 μM were comparable to those of ascorbic acid (50 μM) in both DPPH and ABTS assays (Figure 2a,b left panels).

Since DPPH and ABTS assays are methods used to test radical scavenging in a cell-free system, the inhibitory effect of EGCG on intracellular ROS generation was also evaluated. EGCG-5′Glu was pretreated on RAW264.7 cells, and then SNP was added to induce ROS production. In Figure 2c, EGCG-5′Glu suppressed generation of intracellular ROS by fluorescence-activated cell sorting (FACS) analysis, while ascorbic acid did not strongly suppress ROS generation. EGCG-5′Glu did not display cytotoxicity at 0–25 μM in RAW264.7 cells (Figure 2d). These results indicate that EGCG-5′Glu scavenged both extracellular and intracellular radicals.

### 2.2. Cell Protective Effect of EGCG-5′Glu from SNP-Induced Radicals

**Figure 3:**
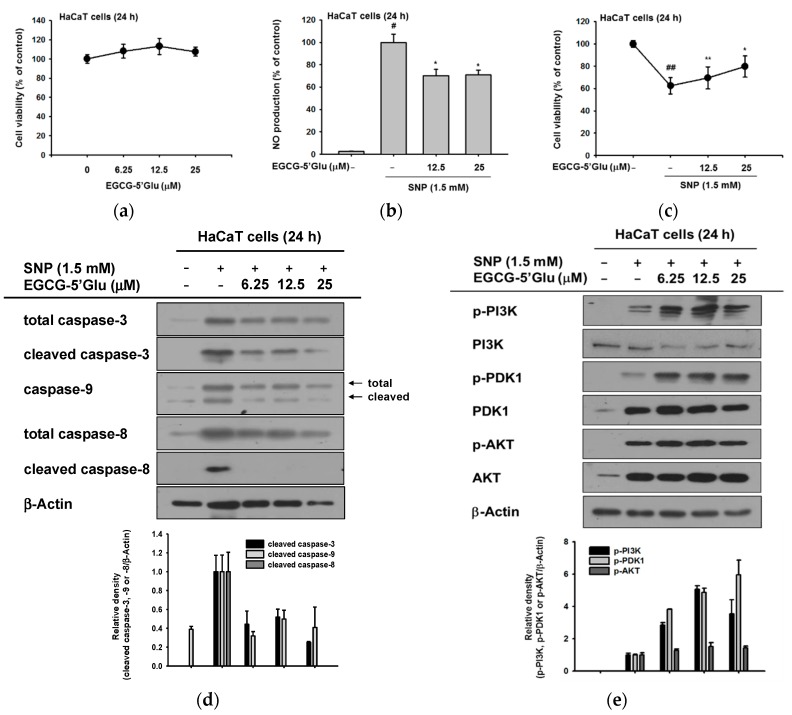
Anti-apoptotic effect of EGCG-5′Glu under SNP-induced apoptosis. (**a**) EGCG-5′Glu was applied to HaCaT cells for 24 h. Cell viability was tested by MTT assay. (**b**) EGCG-5′Glu was pre-treated on RAW264.7 cells for 30 min, and SNP (1.5 mM) was added for 24 h. SNP-derived NO was measured by Griess assay. (**c**) Under SNP treatment, cell viability of HaCaT cells with or without EGCG-5′Glu was identified by MTT assay. (**d**) Caspase levels of EGCG-5′Glu and SNP-treated HaCaT cells were analyzed by immunoblotting. Antibodies against total or cleaved caspase-3, -8, and -9 and β-actin were used. (**e**) Phosphorylated levels of PI3K, PDK1, and AKT in EGCG-5′Glu- and SNP-treated HaCaT cells were analyzed by immunoblotting. Antibodies against phospho- or total forms PI3K, PDK1, AKT, and β-actin were used. # *p* < 0.05 and ## *p* < 0.01 versus a normal group (untreated group), * *p* < 0.05 and ** *p* < 0.01 versus a control group (SNP-treated group).

Next, we aimed to investigate the protective effect of EGCG-5′Glu from radicals in skin. Constant exposure to UV irradiation or chemical substances induces generation of free radicals or ROS in skin. These radicals cause collagen disruption and skin damage. Antioxidants scavenge radicals and protect skin from oxidative damage [1,2]. The cell viability of EGCG-5′Glu on keratinocyte cell line HaCaT was tested by 3-(4,5-dimethylthiazol-2-yl)-2,5-diphenyltetrazolium bromide (MTT) assay, and it revealed that EGCG-5′Glu has no toxicity on HaCaT cell line (Figure 3a). ROS inducer SNP and EGCG-5′Glu were applied to HaCaT cells, and nitric oxide (NO) production and cell viability were evaluated. EGCG-5′Glu reduced SNP-generated NO and recovered SNP-induced cell death (Figure 3b,c). These results imply that the antioxidant potential of EGCG-5′Glu cleared ROS and protected cells from ROS. 

Antioxidative EGCG-5′Glu increased cell viability under SNP treated conditions (Figure 3c). It is well known that decrease in ROS with antioxidant treatment suppresses apoptosis [36,37]. We explored the apoptotic signal pathway to decipher the regulatory mechanism of EGCG-5′Glu (Figure 3d). The final effector molecule, caspase-3, was detected by immunoblotting, and its cleaved form was significantly reduced by EGCG-5′Glu. When upstream molecules, caspase-8 and caspase-9, were also detected, the formation of cleaved forms of both molecules was inhibited. These results showed that EGCG-5′Glu could regulate the intrinsic and extrinsic apoptotic pathways together. 

Since EGCG-5′Glu suppressed cleaved caspase-9 formation, the cell survival pathway was verified. Phosphorylated PI3K and PDK1 were dramatically increased when EGCG-5′Glu was treated with SNP (Figure 3e). By regulating the PI3K/PDK1 pathway, the cell survival rate was increased against ROS-mediated apoptosis. 

### 2.3. Cytoprotective Effect of EGCG-5′Glu Against UVB-Induced Damage

**Figure 4:**
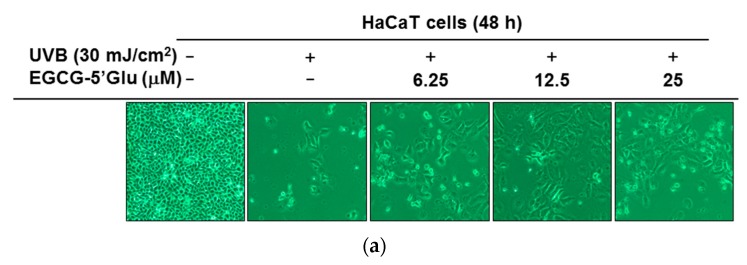

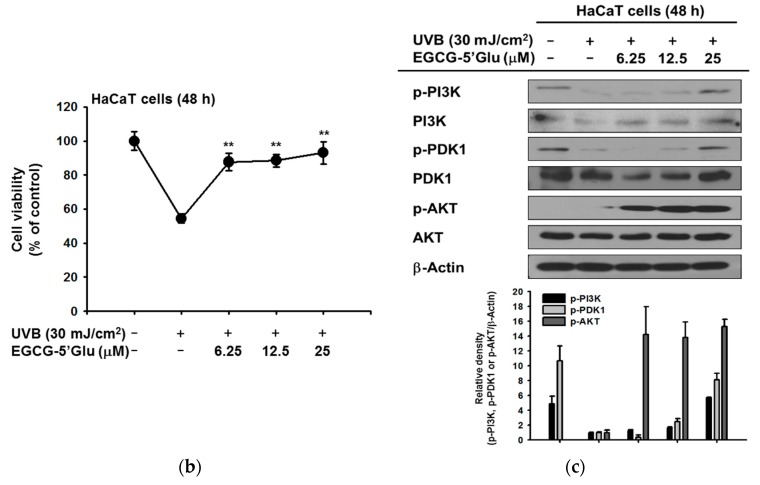
Anti-oxidant effect of EGCG-5′Glu against UVB-induced damage. (**a**) Images of HaCaT cells treated with EGCG-5′Glu (0–25 μM) and UVB (30 mJ/cm^2^) irradiation for 48 h were captured with a camera attached to the microscope. (**b**) Under UVB irradiation, viability of HaCaT cells with and without EGCG-5′Glu was measured by MTT assay. (**c**) Under UVB irradiation, HaCaT cell were incubated with EGCG-5′Glu for 48 h. Phospho- and total PI3K, AKT, and PDK1 expression was detected by immunoblotting. β-Actin was used as an immunoblotting loading control. ** *p* < 0.01 versus a control group (UVB-treated group).

The protective effect of EGCG-5′Glu on chemical substance-induced apoptosis was tested in Figure 3. Next, the cytoprotective effect of EGCG-5′Glu on keratinocytes damaged by ultraviolet B (UVB) was investigated. HaCaT cells were irradiated with 30 mJ/cm^2^ of UVB. Images of HaCaT cells treated with EGCG-5′Glu (0–25 μM) under UVB irradiation were obtained. A large number of UVB-irradiated HaCaT cells were dead; however, EGCG-5′Glu-treated cells showed better survival than only UVB irradiated cells (Figure 4a). Therefore, MTT assay was conducted to confirm the viability of HaCaT cells with EGCG-5′Glu after UVB irradiation. EGCG-5′Glu inhibited cell death caused by UVB irradiation. The cell viability of the group was 54.4% (UVB irradiation), 87.7% (UVB + EGCG-5′Glu 6.25 μM), 88.5% (UVB + EGCG-5′Glu 12.5 μM), and 93% (UVB + EGCG-5′Glu 25 μM) compared to that of the normal group (Figure 4b). Immunoblotting was performed to determine the mechanism by which EGCG-5′Glu enhances cell survival in HaCaT cells under UVB irradiation. The levels of phospho-PI3K, PDK1, and AKT increased in the group treated with EGCG-5′Glu (0–25 μM). When treated with 25 μM of EGCG-5′Glu, the expression levels of phospho-PI3K and PDK1 were similar to the normal level (Figure 4c). These results demonstrate that EGCG-5′Glu can protect HaCaT cells from UVB damages.

### 2.4. Cell Proliferative Effect of EGCG-5′Glu

**Figure 5:**
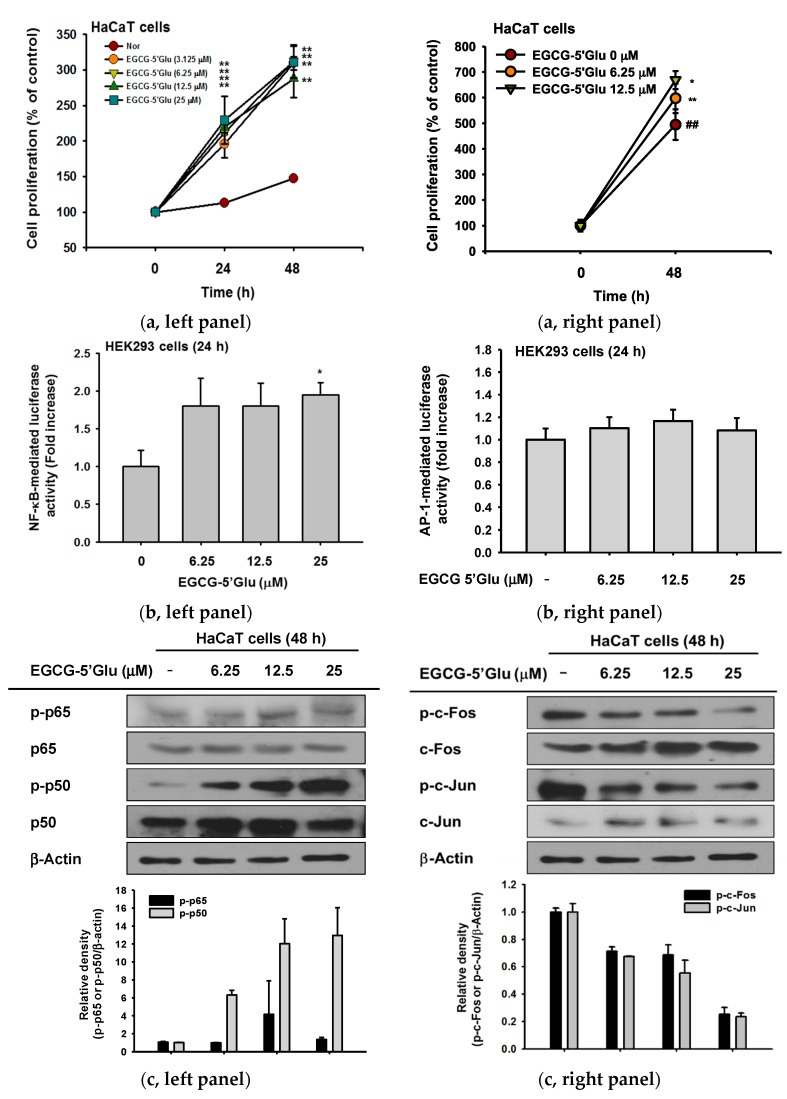

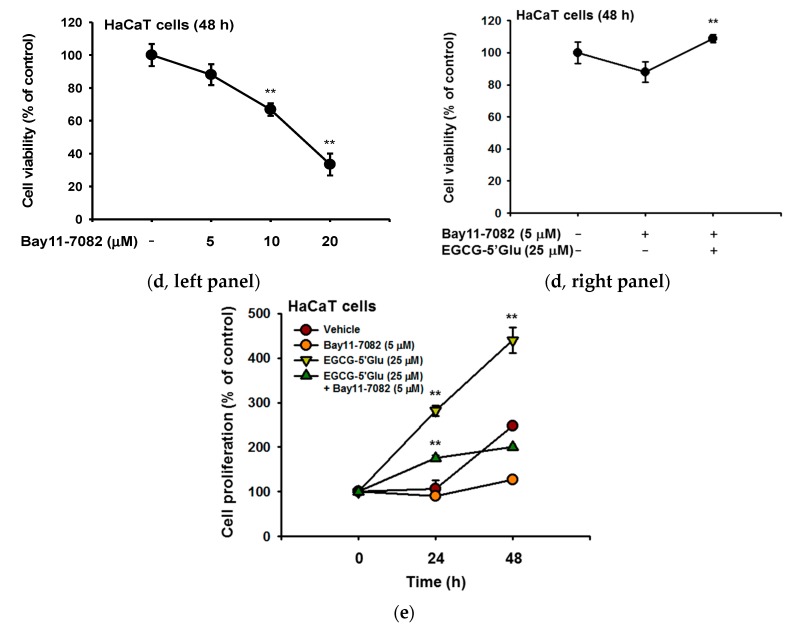
Effect of EGCG-5′Glu on cell proliferation. (**a**) Proliferation of HaCaT cells treated with EGCG-5′Glu (0–25 μM) for 0–48 h was measured by MTT assay (**left panel**) and by Trypan blue dye exclusion assay. (**b**) HEK293 cells were transfected with NF-κB-Luc (**left panel**), AP-1-Luc (**right panel**), and β-gal plasmids and treated with EGCG-5′Glu (0-25 μM) for 24 h. (**c**) Levels of phospho- and total forms of p65 and p50 (**left panel**) and c-Jun and c-Fos (**right panel**) in whole cell lysates were determined by immunoblot analysis after treating HaCaT cells with EGCG-5′Glu (0–25 μM) for 48 h. (**d, left panel**) Viability of Bay11-7082-treated HaCaT cells was measured by MTT assay for 48 h. (**d, right panel**) Bay11-7082 (5 μM) was treated with or without EGCG-5′Glu (25 μM), and viability of HaCaT cells was measured by MTT assay. (**e**) With EGCG-5′Glu (25 μM) treatment, the effect of NF-κB on cell proliferation using Bay11-7082 (5 μM) was confirmed by MTT assay. * *p* < 0.05 and ** *p* < 0.01 versus a control group (normal group).

Previous data have confirmed that EGCG-5′Glu aids cell growth of UVB-irradiated HaCaT cells. Next, a cell proliferation assay was conducted to investigate improvement in skin wrinkles with EGCG-5′Glu. Increased proliferation of cells has been thought a strategy of anti-wrinkle [38,39]. With EGCG-5′Glu treatment, cell proliferation compared to the normal group increased 309.2% (EGCG-5′Glu 3.125 μM), 312.2% (EGCG-5′Glu 6.25 μM), 288% (EGCG-5′Glu 12.5 μM), and 311.1% (EGCG-5′Glu 25 μM) at 48 h (Figure 5a, left panel). To confirm such an increasing pattern, we also carried out counting viable cells using Trypan blue dye exclusion assay. As shown in Figure 5a, the right panel showed, EGCG-5′Glu (6.25 and 12.5 μM) significantly increased the number of cells. Because cell proliferation was increased with EGCG-5′Glu treatment, a reporter gene assay was next performed to investigate the mechanism. NF-κB luciferase activity increased 1.8 fold (EGCG-5′Glu 6.25 μM and 12.5 μM) and 1.95 fold (EGCG-5′Glu 25 μM) (Figure 5b). Immunoblotting was performed to determine the effect of EGCG-5′Glu on NF-κB and AP-1 signaling. In HaCaT cells, treatment with EGCG-5′Glu dramatically increased the level of phospho-p50 in a dose-dependent manner. p65 activity was slightly regulated (Figure 5c, left panel), but the levels of phospho- c-Jun and c-Fos were not affected (Figure 5c, right panel). To prove the effect of NF-κB on cell proliferation, Bay11-7082 was used as an NF-κB inhibitor. The cytotoxicity of Bay11-7082 was confirmed in HaCaT cells (Figure 5d, left panel), and 5 μM Bay11-7082 was used in subsequent experiments. When EGCG-5′Glu was treated with Bay11-7082, cell viability was higher than in the NF-κB alone group (Figure 5d, right panel). Cell proliferation rate for 48 h was also higher with Bay11-7082 and EGCG-5′Glu than with Bay11-7082 alone (Figure 5e). These results demonstrated that EGCG-5′Glu regulates cell proliferation and has ability to improve skin wrinkle formation.

## 3. Discussion

In this study, we synthesized the novel EGCG derivative EGCG-5′Glu and examined its physiological activities. EGCG-5′Glu has antioxidant effects in both cells and cell-free systems. Moreover, we investigated the mechanism by which EGCG-5′Glu protects cells from SNP- or UVB- derived ROS generation. EGCG-5′Glu decreased cleaved caspases and upregulated cell survival molecules.

As outdoor activities have increased in popularity, the skin has become more exposed to UV irradiation and environmental pollution. Among different wavelengths of UV, UVB can cause multiple skin diseases including skin cancer because it directly damages DNA. Free radicals and ROS are also increased with UVB. High levels of free radicals and ROS can cause skin damages, inflammation, or cancer. Therefore, the clearing of free radicals is important to maintain healthy bodies. In our results, EGCG-5′Glu removed various radicals. Moreover, EGCG-5′Glu increased HaCaT cell viability by 38.6% under UVB irradiation (Figure 4a,b). Elements of the PI3K/PDK1/AKT pathway—a cell protection mechanism—were identified by immunoblotting [40]. EGCG-5′Glu has antioxidative properties, and its regulatory mechanism was revealed. In vivo and clinical trials are required for further study, but our data suggest that EGCG-5′Glu may have utility as a dietary supplement or cosmetic ingredient. 

In addition to protecting cells from radicals, EGCG-5′Glu also regulated cell survival. When HaCaT cells were treated with EGCG-5′Glu to measure the cell proliferative rate, cells proliferated by 164.6% compared with the normal group in up to 48 h (Figure 5a). In addition, EGCG-5′Glu regulated cell proliferation through the NF-κB pathway, as demonstrated by luciferase reporter gene assay and immunoblotting (Figure 5b,c). Bay11-7082 (NF-κB inhibitor) [41] was used to confirm that EGCG-5′Glu is regulated via NF-κB signaling (Figure 5d,e). Together with an antioxidant effect, EGCG-5′Glu could protect and recover cells from harmful factors. Increased proliferation is related to epidermal thickness, and maintenance of the epidermis can block wrinkle formation [42]. Therefore, increased proliferation could be considered an anti-aging action.

EGCG has been studied for its potential antioxidant, anti-inflammation, and anti-apoptosis effects [24,25,26]. Though EGCG has potential utility, EGCG also involves problems such as low water solubility, rapid metabolism, and instability in aqueous solution [23,27,28]. To improve these problems, many researchers have chemically modified EGCG [28,29,30,31]. These derivatives are more active or show increased solubility and stability compared to EGCG. EGCG-5′Glu showed high solubility and browning resistance compared to EGCG [32]. EGCG-5′Glu exhibited antioxidant effects. The cytoprotective effect from UVB irradiation was preferable to that of EGCG, and the regulatory mechanism of apoptosis was different compared to our previous study [26]. By modifying EGCG residue, the problems of EGCG were overcome, and the effect of EGCG-5′Glu was akin to that of EGCG. Glycosylation of EGCG has been considered important for use in food, pharmaceuticals, and cosmetics [32]. These points should facilitate the industrial utilization of EGCG-5′Glu.

**Figure 6:**
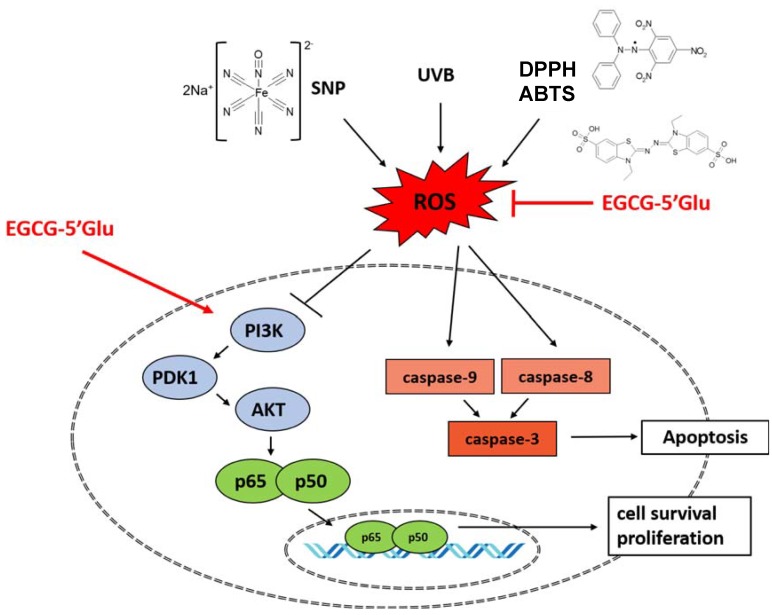
Summary of the cytoprotective effect of EGCG-5′Glu. EGCG-5′Glu cleared various free radicals and downregulated caspase activities. Survival signal pathway (PI3K/AKT/NF-κB) improved with EGCG-5′Glu, and cell proliferation increased. → stimulation, ⊥ inhibition.

In summary, we found that EGCG-5′Glu can protect keratinocytes from various external environments that generate ROS, such as UVB and SNP. EGCG-5′Glu was effective in promoting cell proliferation by increasing survival signaling composed of PI3K and PDK1, which could be used to improve skin wrinkles as the number of cells increases in the dermis layer, as summarized in Figure 6.

## 4. Materials and Methods

### 4.1. Reagents

EGCG-5′Glu was obtained from AmorePacific Co. (Yongin, Korea), as reported previously [32]. Cell lines (HaCaT, HEK293, and RAW264.7 cells) were purchased from American Type Culture Collection (Rockville, MD, USA). Dulbecco’s modified Eagle’s medium (DMEM), RPMI1640, and penicillin-streptomycin were purchased from HyClone (Logan, UT, USA). Fetal bovine serum (FBS) and phosphate buffer saline (PBS) were obtained from Biotechnics Research (Lake Forest, CA, USA) and Capricorn Scientific (Ebsdorfergrund, Germany), respectively. 3-(4-5-Dimethylthiazol-2-yl)-2,5-diphenyltetrazolium bromide (MTT) was purchased from Amresco (Solon, OH, USA). Polyethylenimine (PEI), 1-diphenyl-2-picryl-hydrazyl (DPPH), 2,2′-azino-bis(3-ethylbenzothiazoline-6-sulphonic acid) diammonium salt (ABTS), ascorbic acid, sodium nitroprusside (SNP), dehydrorhodamine 123 (DHR123), and Bay11-7082 were purchased from Sigma Aldrich Chemical Co. (St. Louis, MO, USA). The luciferase assay system was purchased from Promega (Madison, WI, USA). Polyvinylidenedifluoride (PVDF) membrane was purchased from Merck Millipore (Billerica, MA, USA). Antibodies against cleaved or total forms of caspase-3, caspase-8, and caspase-9, and phospho- or total forms of PI3K, PDK1, AKT, p65, p50, c-Jun, c-Fos, and β-actin were obtained from Cell Signaling Technology (Beverly, MA, USA). 

### 4.2. Cell Culture

HaCaT cells were cultured in DMEM with 10% FBS and 1% penicillin-streptomycin at 37 °C in a 5% CO_2_ humidified incubator.

### 4.3. Cell Viability and Cell Proliferation Assay

HaCaT cells were seeded at 3.5 × 10^4^ cells per well in 96-well plates and then treated with EGCG-5′Glu for 24 h. For cell proliferation measurements, HaCaT cells were seeded at 3 × 10^3^ cells per well in 96-well plates and then treated with EGCG-5′Glu (0–25 μM) for 24 h and 48 h. After incubation times of 24 h and 48 h, cell viability and proliferation were measured using conventional MTT assay and trypan blue dye exclusion assay, as reported previously [43,44].

### 4.4. DPPH assay

DPPH decolorimetric assay was performed to examine the scavenging effect of EGCG-5′Glu and EGCG [45,46]. A total of 250 μM DPPH in methanol was prepared. EGCG-5′Glu (0-25 μM) was added to 495 μL DPPH and incubated at 37 °C for 30 min. Ascorbic acid (50 μM) was used as a positive control. After reaction, the absorbance of each fraction at 517 nm was measured by spectrophotometry. DPPH scavenging effect was expressed as percent of inhibition:

DPPH scavenging effect (%) = [(A_0_ − A_1_)/A_0_] × 100% (1)

A_0_ (absorbance of DPPH), A_1_ (absorbance of samples).

### 4.5. ABTS Assay

ABTS scavenging assay was implemented as reported previously [47]. A mixture of 7.4 mM ABTS and 2.4 mM potassium persulfate at a ratio of 1:1 was incubated at room temperature overnight to generate ABTS radical cations (ABTS•+). ABTS solution 100 μL was transferred to a 96-well plate, and EGCG-5′Glu (0–25 μM), EGCG (0–25 μM) or ascorbic acid (50 μM) was added to each well. After a 30 min incubation at 37 °C, the absorbance of the mixture was measured at 730 nm. ABTS scavenging effect was expressed as percentage:

ABTS scavenging effect (%) = [(A_0_ − A_1_)/A_0_] × 100% (2)

A_0_ (absorbance of ABTS), A_1_ (absorbance of samples).

### 4.6. ROS Generation

The level of intracellular ROS was determined by changes in fluorescence resulting from oxidation of the DHR123 fluorescent probe. Briefly, 1 × 10^6^ RAW264.7 cells were exposed to EGCG-5′Glu for 30 min and then incubated with SNP (0.25 mM) at 37 °C for 20 min to induce ROS production. Cells were further incubated with 20 μM of the fluorescent probe DHR123 for 30 min at 37 °C. Ascorbic acid (0–50 μM) was used as a positive control. The degree of fluorescence, which corresponds to the level of intracellular ROS, was determined using a FACScan flow cytometer (Becton-Dickinson, San Jose, CA, USA), as reported previously [48,49]. 

### 4.7. NO Production and Griess Assay

After preincubation for 18 h, HaCaT cells (4 × 10^5^ cells/mL) were pre-treated with EGCG-5′Glu (0–25 μM) for 30 min, followed by incubation with SNP (1.5 mM) for 24 h. The inhibitory effects of EGCG-5′Glu on SNP-induced NO production were determined using the Griess reagent to measure NO level, as previously described [50].

### 4.8. Immunoblot Assay

EGCG-5′Glu-treated cells were washed in PBS. Cells were then pelleted and lysed in cell lysis buffer for 1 h at 4 °C. Protein lysate was pelleted using a centrifuge (12,000 rpm, 10 min, 4 °C). Supernatant concentrations were then measured using Bradford assay, and the concentration was adjusted between samples. Proteins were analyzed using immunoblotting. Phospho- or total levels of PI3K, PDK1, AKT, p50, p65, c-Fos, c-Jun, and caspase-3, -8, and -9 were assessed by previously published methods [45,51]. β-Actin was used as an immunoblotting loading control.

### 4.9. UVB Irradiation

HaCaT cells were seeded at 7 × 10^5^ cells per well in 6-well plates and incubated to 24 h of starvation using serum-free minimal essential medium (MEM). Before UVB irradiation, HaCaT cells were pretreated with EGCG-5′Glu for 30 min. Then, HaCaT cells were washed with DPBS and exposed to 30 mJ/cm^2^ of UVB irradiation (UVB lamp: Bio-link crosslinker (BLX)-312; Vilber Lourmat, Collegien, France). After UVB irradiation, DMEM medium containing EGCG-5′Glu (0–25 μM) was added to cells and incubated for 24 h.

### 4.10. Reporter Gene Assay

HEK293 cells were seeded at 1 × 10^4^ cells per well in 24-well plates. After 24 h, cells were transfected with NF-κB-Luc, AP-1-Luc, and β-galactosidase with media not containing antibiotics for 24 h. PEI was used as transfection reagent. Cells were treated with EGCG-5′Glu (0–25 μM) for 24 h. Luciferase assay was conducted using the Luciferase Assay System (Promega, Madison, WI, USA).

### 4.11. Statistical Analysis

All data of this study are expressed as means ± standard deviations (SDs) of an experiment performed with six technical replicates per group for cellular experiments, and three technical replicates per group for biochemical experiments including immunoblotting analysis. For statistical comparison, results were analyzed by ANOVA with Scheffe’s post hoc test, Kruskal-Wallis and Mann-Whitney U tests. For all analyses, *p* < 0.05 was considered statistically significant. All statistical tests were performed with SPSS software (SPSS Inc., Chicago, IL, USA). Similar experimental data were also observed using an additional independent set of experiments that was conducted using the same numbers of samples.
